# Effects of Global Warming on Ancient Mammalian Communities and Their Environments

**DOI:** 10.1371/journal.pone.0005750

**Published:** 2009-06-03

**Authors:** Larisa R. G. DeSantis, Robert S. Feranec, Bruce J. MacFadden

**Affiliations:** 1 Department of Biology, University of Florida, Gainesville, Florida, United States of America; 2 Division of Vertebrate Paleontology, Florida Museum of Natural History, Gainesville, Florida, United States of America; 3 New York State Museum, Albany, New York, United States of America; Umea University, Sweden

## Abstract

**Background:**

Current global warming affects the composition and dynamics of mammalian communities and can increase extinction risk; however, long-term effects of warming on mammals are less understood. Dietary reconstructions inferred from stable isotopes of fossil herbivorous mammalian tooth enamel document environmental and climatic changes in ancient ecosystems, including C_3_/C_4_ transitions and relative seasonality.

**Methodology/Principal Findings:**

Here, we use stable carbon and oxygen isotopes preserved in fossil teeth to document the magnitude of mammalian dietary shifts and ancient floral change during geologically documented glacial and interglacial periods during the Pliocene (∼1.9 million years ago) and Pleistocene (∼1.3 million years ago) in Florida. Stable isotope data demonstrate increased aridity, increased C_4_ grass consumption, inter-faunal dietary partitioning, increased isotopic niche breadth of mixed feeders, niche partitioning of phylogenetically similar taxa, and differences in relative seasonality with warming.

**Conclusion/Significance:**

Our data show that global warming resulted in dramatic vegetation and dietary changes even at lower latitudes (∼28°N). Our results also question the use of models that predict the long term decline and extinction of species based on the assumption that niches are conserved over time. These findings have immediate relevance to clarifying possible biotic responses to current global warming in modern ecosystems.

## Introduction

Recent global warming alters species distributions, abundances, interactions, and the timing of seasonal activities [1]–[3]. Bioclimatic ‘envelope’ models examining current warming trends predict the long term decline and extinction of species. Generally, these models are based on an understanding of the modern ecological parameters of species, and often incorporate an assumption of niche conservatism, i.e. the idea that ecological niches are maintained over long time scales [4]–[6]. The fossil record provides a long-term record from which the effects of past global warming can be assessed. Previous work comparing mammalian communities during the last ∼780,000 years has documented only minor declines in small mammal species richness with increased warming [7]. Dietary reconstructions inferred from stable isotopes of mammalian tooth enamel yield minor to no changes between glacial and interglacial periods in both large and small mammals [8], [9]. Similarly, there is evidence that the niches of mammalian taxa, based on temperature and precipitation, that persisted during the last glacial to interglacial transition, are conserved [10]. All of these studies suggest that mammalian responses to interglacial warming were generally minor.

Here we present stable carbon and oxygen isotope data of medium to large mammals from a glacial and an interglacial site in Florida. We first compare carbon isotope values and evaluate the hypothesis that dietary niches, inferred from the mean and breadth of carbon isotope values, did not change with interglacial warming. Next, carbon and oxygen isotope data from serially-sampled horse teeth are used to elucidate if and how relative seasonality differed between the glacial and interglacial period, with oxygen isotope data from all taxa documenting changes in relative humidity [11]. This study tests how interglacial warming affected mammalian diets as well as documents the magnitude of climatic differences at these low latitude (∼28°N) glacial and interglacial sites.

Stable carbon and oxygen isotopes are incorporated into the tooth enamel of mammalian taxa and are representative, respectively, of the food and water consumed while alive. Furthermore, C_3_ plants (e.g. trees, shrubs, and cool season grasses) photosynthesize differently from C_4_ plants (e.g. warm season grasses) and subsequently reflect distinctly lower δ^13^C ranges [12]. Taking into account the ^13^C enrichment from food to tooth enamel (∼14‰) as well as the decline in δ^13^C values (∼1.5‰) of atmospheric CO_2_ due to fossil fuel burning over the past two centuries [13]–[15], tooth enamel values of less than −8‰ indicate a diet consisting of primarily C_3_ vegetation whereas δ^13^C values of greater than −2‰ indicate a diet of predominantly C_4_ vegetation [12], [14]. Additionally, C_3_ grasses are a rare or absent component of the landscape in Florida during the last ∼2 million years [16]–[19], further enabling δ^13^C values of less than −8‰ to indicate a predominantly browsing diet. Lower δ^13^C values can also indicate the consumption of browse in denser canopied C_3_ forests [20]–[22]. By comparing stable carbon isotope values between individuals and populations during a glacial and an interglacial period, we can assess how long-term warming affected mammalian diets. Specifically, we compare the relative consumption of C_3_ browse vs. C_4_ grass, as inferred from δ^13^C values. In addition, oxygen isotopes in mammalian tooth enamel typically document changes in temperature and precipitation, with ^18^O enrichment indicating a warmer and/or drier climate [23]–[25]. Additionally, when comparing the oxygen isotope values of mammals that obtain a large proportion of their water from plants, relative aridity can be assessed [11], [25].

Based on the depth of terrestrial fossils in relation to current sea-levels, the coastal fossil sites of Inglis 1A and Leisey 1A (Figure S1) represent a glacial and an interglacial site, respectively. Inglis 1A (29°0′N, 82°41′W; ∼2.0–1.6 Ma) exhibits terrestrial fossils within a sinkhole in the Eocene Inglis Formation at depths of 5 meters below sea-level and completely lacks a marine fauna, indicating lower sea-levels that would have occurred during a glacial period [26]. This glacial interpretation is also supported by the presence of cooler adapted taxa such as the muskrat *Ondatra* cf. *idahoensis* and pronghorn *Capromeryx arizonensis* [26]. The younger Leisey 1A (27°42′N, 82°30′W; ∼1.6–1.3 Ma) locality instead has terrestrial and marine fossils intermixed between two shell beds at depths consistent with interglacial levels [26]. Leisey 1A also has taxa indicative of a warmer interglacial period including alligators (*Alligator mississippiensis* [26]). These geographically similar terrestrial localities provide the rare opportunity to examine how mammals altered their diets in response to interglacial warming during the late Pliocene to early Pleistocene.

All mammalian taxa present within the orders of Artiodactyla, Perissodactyla, and Proboscidea were sampled for enamel stable carbon and oxygen isotopes (n = 115; Table S1 and S2; see supporting information and *Materials and Methods*). Representing 8 families, these taxa include the following: deer (*Odocoileus virginianus*), horses (*Equus* sp.), llamas (*Hemiauchenia macrocephala*, *Palaeolama mirifica*), peccaries (*Mylohyus fossilis*, *Platygonus vetus*), proboscideans (*Cuvieronius tropicus*, *Mammut americanum*, *Mammuthus hayi*), pronghorn (*Capromeryx arizonensis*), and tapirs (*Tapirus* sp., including *Tapirus haysii*). These mammals were compared both within (Table 1 and 2) and between fossil localities to demonstrate how dietary resources were partitioned. Serial samples (samples taken at a series of intervals perpendicular to the growth axis of the tooth) of the high-crowned horses were also taken from specimens at both localities, enabling comparisons of relative seasonality (*n* = 23; Table S3).

**Table 1:** Significant differences in δ^13^C values among taxa from Inglis 1A, Florida.

Inglis 1A, Florida	*Tapirus* sp.	*Capromeryx arizonensis*	*Mammut americanum*	*Platygonus vetus*	*Hemiauchenia macrocephala*	*Equus* sp.
*Odocoileus virginianus*	**0.045**	**0.027**	0.063	***p*** **<0.0001**	***p*** **<0.0001**	***p*** **<0.0001**
*Tapirus* sp.		0.447	0.438	**0.010**	**0.002**	***p*** **<0.0001**
*Capromeryx arizonensis*			0.862	0.255	0.092	***p*** **<0.0001**
*Mammut americanum*				0.516	0.279	***p*** **<0.0001**
*Platygonus vetus*					0.339	***p*** **<0.0001**
*Hemiauchenia macrocephala*						***p*** **<0.0001**

Bold *p*-values indicate significant differences.

**Table 2:** Significant differences in δ^13^C values among taxa from Leisey 1A, Florida.

Leisey 1A, Florida	*Tapirus haysii*	*Mammut americanum*	*Odocoileus virginianus*	*Mylohyus fossilis*	*Hemiauchenia macrocephala*	*Platygonus vetus*	*Cuvieronius tropicus*	*Equus* sp.	*Mammuthus hayi*
*Palaeolama mirifica*	0.711	0.284	0.082	***p*** **<0.0001**	***p*** **<0.0001**	***p*** **<0.0001**	***p*** **<0.0001**	***p*** **<0.0001**	***p*** **<0.0001**
*Tapirus haysii*		0.415	0.124	***p*** **<0.0001**	***p*** **<0.0001**	***p*** **<0.0001**	***p*** **<0.0001**	***p*** **<0.0001**	***p*** **<0.0001**
*Mammut americanum*			0.578	***p*** **<0.001**	***p*** **<0.0001**	***p*** **<0.0001**	***p*** **<0.0001**	***p*** **<0.0001**	***p*** **<0.0001**
*Odocoileus virginianus*				***p*** **<0.001**	***p*** **<0.0001**	***p*** **<0.0001**	***p*** **<0.0001**	***p*** **<0.0001**	***p*** **<0.0001**
*Mylohyus fossilis*					**0.003**	**0.001**	***p*** **<0.0001**	***p*** **<0.0001**	***p*** **<0.0001**
*Hemiauchenia macrocephala*						0.749	**0.037**	***p*** **<0.0001**	***p*** **<0.0001**
*Platygonus vetus*							0.060	***p*** **<0.0001**	***p*** **<0.0001**
*Cuvieronius tropicus*								0.232	0.183
*Equus* sp.									0.749

Bold *p*-values indicate significant differences.

## Results and Discussion

### Dietary Partitioning

**Figure 1:**
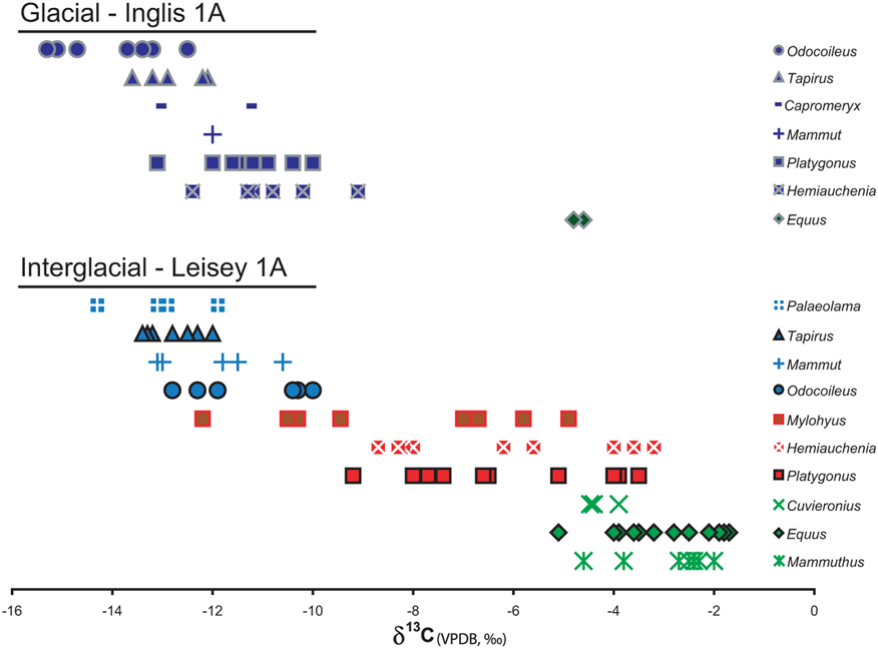
Carbon isotopic niche partitioning of all sampled taxa. Stable carbon isotope values for all taxa sampled at Inglis 1A (I) and Leisey 1A (L), with dominant C_3_ browsers (blue), mixed C_3_ and C_4_ consumers (red), and dominant C_4_ grazers (green) indicated.

The glacial Inglis 1A locality represents a C_3_-dominated community with all taxa, except for *Equus*, having δ^13^C values more negative than −9.1‰ (Figure 1 and S2, Table S1 and S2). As C_3_ grasses are a rare or absent component of Florida landscapes during the last ∼2 million years [16]–[19], all ungulate taxa are interpreted to be C_3_ browsers, with the exception of *Equus*. Resource partitioning within this C_3_-dominated community is apparent with *Odocoileus* δ^13^C values significantly less than all other taxa sampled (with *n*>1) and *Tapirus* significantly less than *Platygonus* and *Hemiauchenia* (Table 1). *Equus* is more enriched in ^13^C than all other taxa (*p*<0.0001), indicating a diet consisting mainly of C_4_ vegetation (i.e. warm season grasses, as C_4_ dicots and CAM vegetation with similar δ^13^C values are rare or absent in Florida [17], [27], [28]). Thus, this glacial site is dominated by C_3_ browsers, although it is clear from the horse data that C_4_ grasses were present. Due to the reliance of the majority of taxa on C_3_ vegetation and the relative rarity of *Equus* at Inglis 1A, we hypothesize that C_4_ grasses likely occurred in much lower abundance.

The interglacial fossil site Leisey 1A consists of taxa which display a large range in δ^13^C values from −14.3 to −2.0‰ (Figure 1 and S2, Table S1 and S2). All genera (with *n*>1) present at both Leisey 1A and Inglis 1A are significantly more enriched in ^13^C at the interglacial site (*Equus*, *p*<0.05; *Hemiauchenia*, *p* = 0.0001; *Odocoileus*, *p* = 0.001; *Platygonus*, *p* = 0.0001) with the exception of *Tapirus*. Increased δ^13^C values in these taxa demonstrates increased inclusion of C_4_ grasses in their diets, although *Odocoileus* may instead be browsing from a more open canopied forest during the interglacial period. The lack of dietary changes in tapirs is consistent with their morphologically and isotopically inferred dense-canopy browsing diet over time [29], [30]. Additionally, the mixed feeders, *Hemiauchenia* and *Platygonus*, increased their isotopic niche breadth from a total δ^13^C range of 3.3‰ and 3.1‰ at the glacial site to 5.5‰ and 5.7‰ at the interglacial site, respectively (Figure 1, Table S1). Leisey 1A potentially has increased ungulate diversity compared to Inglis 1A with the addition of the peccary (*Mylohyus fossilis*), the llama (*Palaeolama mirifica*), and two proboscideans (*Cuvieronius tropicus*, *Mammuthus hayi*), but lacks the pronghorn (*Capromeryx arizonensis*). However, Inglis 1A (∼7700) has fewer specimens than Leisey 1A (∼22,000) which may bias diversity estimates due to taphonomic processes. Both peccary taxa and both llama taxa show significantly different values. *Mylohyus* has lower δ^13^C values than *Platygonus* (*p*<0.05; Figure 1, Table 2 and S1). Similarly, *Palaeolama* has low δ^13^C values as compared to *Hemiauchenia* (*p*<0.0001; Figure 1, Table 2 and S1). Although the proboscideans cannot be statistically compared across sites due to limited sample size at Inglis 1A, the isotopically inferred C_4_ grazing proboscideans (*Cuvieronius*, *Mammuthus*) are only present at Leisey 1A while the browsing mastodon (*Mammut*) is found at both localities. Because *Mammuthus* has one of its earliest occurrences in North America from Leisey 1A, it was absent from the older Inglis 1A locality regardless of ecological factors. However, *Cuvieronius* was present in the late Pliocene of North America [31] and could have been present at Inglis 1A; thus, its absence may represent an ecological signal.

During the interglacial period represented by Leisey 1A, dietary resource use by the majority of ungulate taxa were significantly different from each other (Table 2). However, the isotopically inferred browsers (*Palaeolama*, *Tapirus*, *Mammut*, and *Odocoileus*) lacked significant differences when compared to each other (Table 2). Similarly, the isotopically inferred grazers (*Cuvieronius*, *Equus*, and *Mammuthus*) lacked significant differences from one another (Table 2). This high degree of dietary niche partitioning among the Leisey 1A mammalian community, especially among taxa within the same family, may contribute to its higher diversity (Table 2 and S1). For example, *Palaeolama* and *Mylohyus* may be able to coexist with phylogenetically similar taxa during the interglacial period because they were able to successfully partition food resources. The increased inclusion of C_4_ grasses by presumed dietary generalists such as *Hemiauchenia* and *Platygonus*, the presence of the C_4_ grazers *Cuvieronius* and *Mammuthus*, and the relative abundance of *Equus* at Leisey 1A likely represents the increased abundance of C_4_ vegetation and the potential expansion of C_4_ grasslands during interglacial periods in Florida. However, despite the increased consumption of C_4_ grasses by ungulates at Leisey 1A, none of the grazers are interpreted to consume only C_4_ grasses based on the presence of individuals with δ^13^C values of <−1.5‰. The lack of obligate ungulate grazers during an interglacial period in Florida is somewhat surprising based on modern analogues and further demonstrates the importance of C_3_ dietary resources for all taxa present during these time periods.

In contrast to our data, Koch et al. (1998) demonstrated minor to no differences in ungulates from a full glacial period to late glacial periods during the Pleistocene. The mastodon (*Mammut americanum*) population from the full glacial West Palm Beach locality (∼25,000 BP) has significantly greater δ^13^C values then at the late glacial Cutler Hammock locality (∼11,000 to 9500 BP [8]). However, the remainder of the taxa lack significant dietary differences as inferred from δ^13^C values [8]. The lack of significant changes in dietary resources may be due to the limited sample sizes of taxa from localities with radiocarbon dates that can be discretely defined as full glacial or late glacial ages. The scope of the Koch et al. (1998) paper was to compare taxa with more specialized diets of either browse or grass; therefore, by excluding potential dietary generalists such as *Platygonus* and/or *Hemiauchenia*, the effects of warming on dietary niches may be less apparent. Lastly, the effects of interglacial warming on mammalian communities and their environments may not have been as profound during the late Pleistocene to the early Holocene, as compared to the late Pliocene to the early Pleistocene.

### Paleoclimate and Seasonality

**Figure 2:**
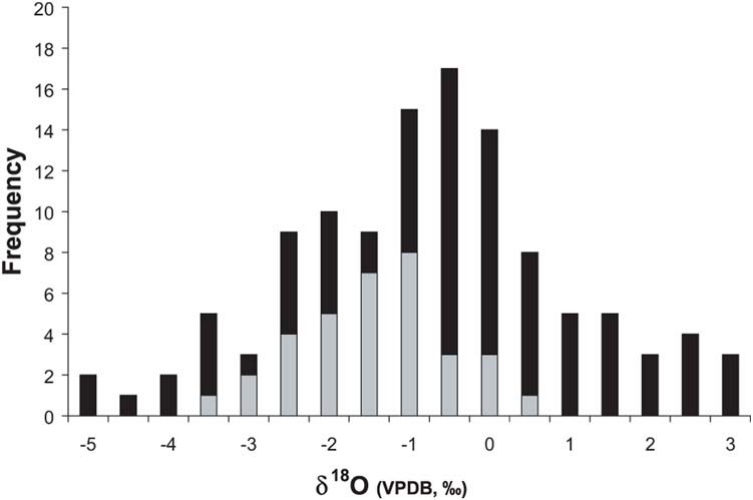
Stacked histogram of oxygen isotope values for all taxa sampled. Frequency of δ^18^O values for taxa from the glacial fossil site Inglis 1A (grey) and from the interglacial fossil site Leisey 1A (black).

Oxygen isotopes of the fossil mammals present at Leisey 1A have a greater range of δ^18^O values than those at Inglis 1A, collectively. Oxygen isotope values range from −3.4 to 0.3‰ at Inglis IA (total range of 3.7‰) and from −5.1 to 2.9‰ at Leisey IA (total range of 8.0‰; Figure 2 and S2, Table S1). For mammals that get the majority of their water from food, δ^18^O values increase with temperature and/or aridity. Based on the aridity index of Levin *et al.* [11], the increased δ^18^O range at Leisey 1A indicates a drier climate (Figure 2 and S2). These data are in agreement with 50,000-year-old pollen records that indicate drying with interglacial warming in Florida [32]. All genera (with *n*>1) present at both localities become significantly more enriched in ^18^O at the interglacial site (*Hemiauchenia*, *p* = 0.0001; *Odocoileus*, *p*<0.05; *Platygonus*, *p*<0.01) with the exception of *Tapirus* and *Equus*, the latter of which lacks statistical significance potentially due to sample size. Conversely, *Tapirus* demonstrates significantly lower δ^18^O values (*p* = 0.0001) during the interglacial. As modern tapirs are observed to have semi-aquatic behavior, the decline in δ^18^O values may indicate an increase in semi-aquatic behavior with warming [33].

**Figure 3:**
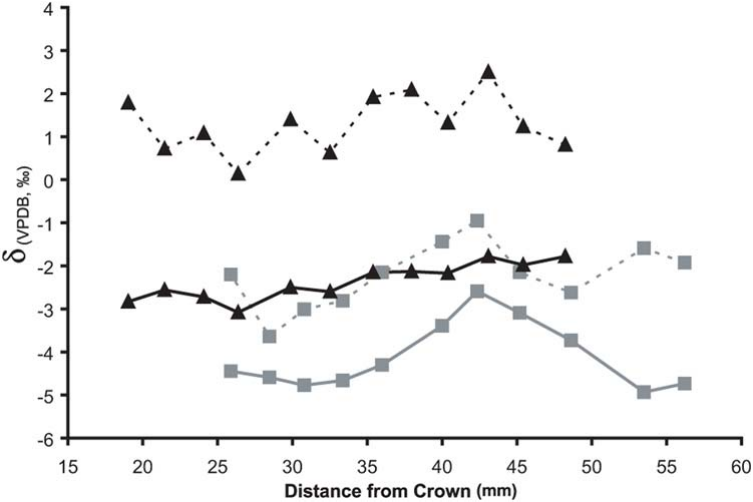
Serial carbon and oxygen isotope samples from fossil horse teeth. Serial carbon (solid lines) and oxygen (dashed lines) isotope samples of horse teeth from the glacial (Inglis 1A, squares) and interglacial (Leisey 1A, triangles) fossil sites.

Serial samples of *Equus* teeth have significantly greater mean δ^13^C and δ^18^O values (*p*<0.0001, for both) at Leisey 1A (δ^13^C = −2.4‰, δ^18^O = 1.3‰) than at Inglis 1A (δ^13^C = −4.1‰, δ^18^O = −2.2‰; Figure 3, Table S3). The serial carbon isotope samples at Inglis 1A indicate greater variability than at Leisey 1A, with total δ^13^C ranges of 2.3‰ and 1.3‰, respectively. Aside from greater δ^13^C variation at Inglis 1A, the δ^13^C values oscillate in a predictable manner that correlates with seasonal warming and cooling (i.e. greater δ^13^C values during the summer and lower δ^13^C values during the winter; Figure 3). This oscillating pattern is likely the result of seasonal variability in the consumption of C_3_ and C_4_ vegetation and/or the ^13^C enrichment and depletion of vegetation due to seasonal water stress [34], [35]. Oxygen isotopes similarly track seasonal variation in temperature and/or precipitation at Inglis 1A. The greater δ^18^O values at Leisey 1A reflect a warmer and/or drier climate than Inglis 1A. Leisey 1A likely experienced a less seasonally predictable climate as δ^18^O values do not fluctuate in a predictable oscillating pattern. Variation in these δ^18^O values may instead be due to precipitation events, with periodic lower δ^18^O values reflecting increased precipitation [23], [24]. Even once patterns of mineralization are accounted for [36]–[38], the pattern of δ^18^O variation at the interglacial Leisey 1A site is similar to patterns of δ^18^O variation in Florida today (Figure S3). These data further support the designations of Inglis 1A and Leisey 1A as a glacial and an interglacial site, respectively, demonstrating that changes in relative seasonality occur with increased warming even at low latitudes of ∼28°N.

### Concluding Remarks

Contrary to previous studies, we document dramatic dietary and floral changes with interglacial warming. The majority of taxa analyzed increase their mean δ^13^C values and/or isotopic niche breadth with warming. Additionally, closely related taxa partitioned their dietary resources differently when sympatric at the interglacial site. Our data falsify the initial hypothesis of niche conservatism, instead showing that increased warming resulted in changes in both the type and breadth of resource use in mammals. Although δ^13^C values reflect only an aspect of an animal's larger dietary niche, significant differences in δ^13^C values as seen here, demonstrates considerable differences in a component of the dietary niches of mammalian taxa. These data imply that models which incorporate data under the assumption of niche conservation may not accurately predict the impacts of global warming on mammalian species. Furthermore, oxygen isotopes in fossil mammal teeth demonstrate increased aridity and decreased relative seasonality with interglacial warming. This study highlights the need for further investigations aimed at understanding paleoecology of species over various time and climatic scales for inferring the future effects of global warming.

## Materials and Methods

A total of 115 specimens were sampled for stable isotopes of tooth enamel, the preferred tissue for geochemical analysis as it reliably reflects original isotopic values [12], [14], [39]. Late erupting teeth (e.g. fourth premolars and third molars) were preferentially selected for sampling when available; however, due to limited sample availability some early erupting teeth and/or fragmentary specimens were sampled. While the stable carbon and oxygen isotope values of early erupting teeth may be influenced by the consumption of the mother's milk, possibly resulting in differences in isotopic values, the early erupting and/or fragmentary teeth sampled here have isotopic values that are within the range of variation of late erupting teeth, and we therefore include these specimens in our analysis (Table S1 and S2). Using a low speed dental-style drill and carbide dental burrs, bulk samples were taken parallel to the growth axis of the tooth while serial samples were taken perpendicular to the growth axis. All enamel powder was pretreated with 30% hydrogen peroxide for 24 hours and 0.1 N acetic acid for 12 hours to remove organics and secondary carbonates, respectively [40]. Approximately 1 mg of these samples were then run on a VG Prism stable isotope ratio mass spectrometer with an in-line ISOCARB automatic sampler in the Department of Geological Sciences at the University of Florida. The analytical precision is ±0.1‰, based on replicate analyses of samples and standards (NBS-19). Stable isotope data were normalized to NBS-19 and are reported in conventional delta (δ) notation for carbon (δ^13^C) and oxygen (δ^18^O), where δ^13^C (parts per mil, ‰) = ((R_sample_/R_standard_)-1)*1000, and R = ^13^C/^12^C; and, δ^18^O (parts per mil, ‰) = ((R_sample_/R_standard_)-1)*1000, and R = ^18^O/^16^O; and the standard is VPDB (Pee Dee Belemnite, Vienna Convention [41]). All stable isotopes are from the carbonate portion of tooth enamel hydroxylapatite.

All carbon and oxygen isotope values within the same locality were analyzed using Fisher's LSD multiple comparisons, as all samples from taxa with adequate sample size had δ^13^C and δ^18^O values that were normally distributed (Shapiro-Wilk tests). When comparing across genera between localities, t-tests were used. T-tests were also used to compare all individual serial samples per tooth, between localities. The aridity index of Levin et al. (2006) was used to test if interglacial warming resulted in increased aridity by comparing the total range of δ^18^O values between localities. By comparing the δ^18^O values of the entire fauna, the total range of δ^18^O values of the most variable evaporation sensitive taxa (i.e. taxa that obtain a large portion of their water from plants) is captured.

## Supporting Information

Table S1 Descriptive statistics of stable carbon and oxygen isotopes from all taxa sampled.
(0.09 MB PDF)

Table S2 All ungulate specimens sampled and their corresponding δ^13^C and δ^18^O values.
(0.10 MB PDF)

Table S3 Serial samples and descriptive statistics of *Equus* sp. teeth from Inglis 1A and Leisey 1A.
(0.09 MB PDF)

Figure S1 Map indicating the location of Inglis 1A and Leisey 1A in Florida, USA.
(0.48 MB JPG)

Figure S2 Stable carbon and oxygen isotope values for all taxa sampled. Blue symbols show taxa from the glacial fossil site Inglis 1A (I) and orange symbols show taxa from the interglacial fossil site Leisey 1A (L). The blue and orange bars indicate the total range of δ^18^O values for Inglis 1A and Leisey 1A, respectively.
(0.31 MB TIF)

Figure S3 Modern climate data from Tampa and Gainesville, Florida, USA. The oxygen isotope data (rainfall precipitation) are from ISOSCAPES (www.waterisotopes.org) and temperature and precipitation data are from the National Climatic Data Center (www.ncdc.noaa.gov), with mean values from the Tampa International Airport and Gainesville Regional Airport during 1971 to 2000.
(1.00 MB TIF)
