# Supplementary material for: Effects of Global Warming on Ancient Mammalian Communities and Their Environments
Source: PLoS One. 2009 Jun 3;4(6):e5750. doi: 10.1371/journal.pone.0005750 (PMC2684586; doi:10.1371/journal.pone.0005750)
Supplement: Table S1 — Descriptive statistics of stable carbon and oxygen isotopes from all taxa sampled. (0.09 MB PDF) [file pone.0005750.s001.pdf]

Table S1. Stable carbon and oxygen isotope values from all taxa sampled from Inglis 1A and Leisey 1A, Florida.

		$\delta^{13}C_{(VPDB)}$ Values (‰)					$\delta^{18}O_{(VPDB)}$ Values (‰)				
Taxa	*n*	Mean	Min.	Max.	Range	SD	Mean	Min.	Max.	Range	SD
**Inglis 1A - Glacial Site**											
Artiodactyla											
Capromeryx arizonensis	2	-12.2	-13.1	-11.3	1.8	1.3	-1.0	-2.1	0.2	2.3	1.6
Hemiauchenia macrocephala	7	-10.9	-12.4	-9.1	3.3	1.0	-1.4	-2.5	-0.8	1.7	0.6
Platygonus vetus	9	-11.4	-13.1	-10.0	3.1	0.9	-2.3	-3.4	-1.5	1.9	0.7
Odocoileus virginianus	8	-13.9	-15.3	-12.5	2.8	1.0	-1.0	-2.3	0.0	2.3	0.8
Perissodactyla											
Equus sp.	2	-4.7	-4.8	-4.6	0.2	0.1	-1.0	-2.2	0.3	2.5	1.8
Tapirus sp.	5	-12.8	-13.6	-12.1	1.5	0.6	-0.8	-1.4	-0.3	1.1	0.4
Proboscidea											
Mammut americanum	1	-12.0	-	-	-	-	-1.2	-	-	-	-
**Leisey 1A - Interglacial Site**											
Artiodactyla											
Hemiauchenia macrocephala	10	-6.4	-8.7	-3.2	5.5	2.2	1.5	-0.8	2.9	3.7	1.2
Palaeolama mirifica	6	-13.0	-14.3	-11.9	2.4	0.8	-0.6	-0.9	-0.2	0.7	0.2
Mylohyus fossilis	9	-8.6	-12.2	-4.9	7.3	2.5	-0.3	-3.5	1.7	3.2	2.5
Platygonus vetus	10	-6.2	-9.2	-3.5	5.7	2.0	0.1	-3.8	2.9	6.7	2.1
Odocoileus virginianus	7	-11.5	-12.8	-10.0	2.8	1.2	0.8	-1.9	2.8	4.7	1.8
Perissodactyla											
Equus sp.	14	-3.1	-5.1	-1.7	3.4	1.0	-0.3	-1.9	1.6	3.5	0.9
Tapirus haysii	9	-12.7	-13.4	-12.0	1.4	0.5	-3.6	-5.1	-2.1	3.0	1.1
Proboscidea											
Cuvieronius tropicus	3	-4.3	-4.5	-3.9	0.6	0.3	0.1	-0.3	0.3	0.6	0.3
Mammut americanum	5	-12.0	-13.1	-10.6	2.5	1.1	-2.3	-3.4	-0.3	3.1	1.2
Mammuthus hayi	8	-2.9	-4.6	-2.0	2.6	0.9	-0.5	-2.5	0.7	3.2	1.2
