# Supplementary material for: Effects of Global Warming on Ancient Mammalian Communities and Their Environments
Source: PLoS One. 2009 Jun 3;4(6):e5750. doi: 10.1371/journal.pone.0005750 (PMC2684586; doi:10.1371/journal.pone.0005750)
Supplement: Table S2 — All ungulate specimens sampled and their corresponding δ13C and δ18O values. (0.10 MB PDF) [file pone.0005750.s002.pdf]

Table S2. All ungulate specimens sampled and their corresponding $\delta^{13}C$ and $\delta^{18}O$ values.

UF Collection #	Taxon	Element	Locality	$\delta^{13}C_{(VPDB)}$ (‰)	$\delta^{18}O_{(VPDB)}$ (‰)
18261	*Capromeryx arizonensis*	RM3	Inglis 1A	-13.1	-2.1
45267	*Capromeryx arizonensis*	LP4	Inglis 1A	-11.3	0.2
176730	*Equus* sp.	LI3	Inglis 1A	-4.8	0.3
217742	*Equus* sp.	lM3	Inglis 1A	-4.6	-2.2
17518	*Hemiauchenia macrocephala*	LP4	Inglis 1A	-10.2	-0.8
17519	*Hemiauchenia macrocephala*	RM1	Inglis 1A	-9.1	-1.2
17522	*Hemiauchenia macrocephala*	rm3	Inglis 1A	-12.4	-1.4
18027	*Hemiauchenia macrocephala*	RM3	Inglis 1A	-11.3	-2.5
18223	*Hemiauchenia macrocephala*	lm3	Inglis 1A	-11.3	-1.8
45284	*Hemiauchenia macrocephala*	lm3	Inglis 1A	-11.2	-0.8
227115	*Hemiauchenia macrocephala*	LM3	Inglis 1A	-10.8	-1.3
18119	*Mammut americanum*	rm3	Inglis 1A	-12.0	-1.2
45022	*Odocoileus virginianus*	rm3	Inglis 1A	-12.5	-0.6
45036	*Odocoileus virginianus*	rm3	Inglis 1A	-13.7	-0.7
227631	*Odocoileus virginianus*	RM1 or 2	Inglis 1A	-15.1	-0.1
227639	*Odocoileus virginianus*	rm3	Inglis 1A	-14.7	-1.8
227640	*Odocoileus virginianus*	rm3	Inglis 1A	-13.4	-0.9
227641	*Odocoileus virginianus*	rm3	Inglis 1A	-13.2	-1.5
227642	*Odocoileus virginianus*	rm3	Inglis 1A	-13.4	0.0
227643	*Odocoileus virginianus*	rm3	Inglis 1A	-15.3	-2.3
18188	*Platygonus vetus*	rm3	Inglis 1A	-11.5	-2.3
18196	*Platygonus vetus*	RM3	Inglis 1A	-12.0	-2.4
18203	*Platygonus vetus*	rm3	Inglis 1A	-10.0	-1.5
45313	*Platygonus vetus*	rm3	Inglis 1A	-10.4	-3.2
60027	*Platygonus vetus*	rm3	Inglis 1A	-11.6	-1.6
60030	*Platygonus vetus*	RM3	Inglis 1A	-13.1	-3.4
176742	*Platygonus vetus*	LM1	Inglis 1A	-11.2	-1.7
227644	*Platygonus vetus*	RM3	Inglis 1A	-10.9	-2.9
227645	*Platygonus vetus*	RM3	Inglis 1A	-11.5	-2.0
18175	*Tapirus* sp.	RM2	Inglis 1A	-12.9	-1.4
18176	*Tapirus* sp.	LI1	Inglis 1A	-13.2	-0.8
115969	*Tapirus* sp.	lp3	Inglis 1A	-12.2	-0.3
115970	*Tapirus* sp.	LP4	Inglis 1A	-12.1	-0.9
176713	*Tapirus* sp.	lower molar	Inglis 1A	-13.6	-0.8
88005	*Cuvieronius tropicus*	partial molar	Leisey 1A	-4.4	0.3
88006	*Cuvieronius tropicus*	partial molar	Leisey 1A	-3.9	0.2
88007	*Cuvieronius tropicus*	partial molar	Leisey 1A	-4.5	-0.3
63876	*Equus* sp.	lm3	Leisey 1A	-5.1	-0.2
63880	*Equus* sp.	lm3	Leisey 1A	-3.6	-0.3

65461	*Equus* sp.	lm3	Leisey 1A	-1.9	0.7
65462	*Equus* sp.	lm3	Leisey 1A	-1.7	0.7
82279	*Equus* sp.	LM3	Leisey 1A	-3.6	-0.3
82883	*Equus* sp.	RM3	Leisey 1A	-4.0	-1.9
83722	*Equus* sp.	RM3	Leisey 1A	-2.8	-1.6
84836	*Equus* sp.	RM3	Leisey 1A	-2.1	-0.2
85735	*Equus* sp.	RM3	Leisey 1A	-1.8	-0.3
85739	*Equus* sp.	RM3	Leisey 1A	-3.2	-0.5
85746	*Equus* sp.	RM3	Leisey 1A	-3.5	-1.2
85753	*Equus* sp.	LM3	Leisey 1A	-2.5	1.6
85761	*Equus* sp.	LM3	Leisey 1A	-3.9	-0.9
86077	*Equus* sp.	rm3	Leisey 1A	-3.6	-0.4
64219	*Hemiauchenia macrocephala*	lm3	Leisey 1A	-8.0	0.2
64315	*Hemiauchenia macrocephala*	LP4	Leisey 1A	-8.3	0.8
80053	*Hemiauchenia macrocephala*	rm3	Leisey 1A	-8.3	1.4
80737	*Hemiauchenia macrocephala*	RP4	Leisey 1A	-8.2	0.9
83964	*Hemiauchenia macrocephala*	RP4	Leisey 1A	-3.6	-0.8
83965	*Hemiauchenia macrocephala*	RM3	Leisey 1A	-6.2	1.9
84239	*Hemiauchenia macrocephala*	LP3	Leisey 1A	-4.0	2.6
85085	*Hemiauchenia macrocephala*	RM	Leisey 1A	-8.7	2.9
132000	*Hemiauchenia macrocephala*	rm3	Leisey 1A	-3.2	2.5
142321	*Hemiauchenia macrocephala*	lm3	Leisey 1A	-5.6	2.1
80004	*Mammut americanum*	partial tooth	Leisey 1A	-13	-3.4
80286	*Mammut americanum*	ldp3	Leisey 1A	-10.6	-0.3
84443	*Mammut americanum*	RdP2	Leisey 1A	-13.1	-3.2
88001	*Mammut americanum*	partial tooth	Leisey 1A	-11.8	-2.1
88003	*Mammut americanum*	partial tooth	Leisey 1A	-11.5	-2.3
67451	*Mammuthus hayi*	M3	Leisey 1A	-4.6	0.2
81707	*Mammuthus hayi*	rm3	Leisey 1A	-2.7	0.7
86137	*Mammuthus hayi*	rm	Leisey 1A	-2.0	0.3
86974	*Mammuthus hayi*	rm3	Leisey 1A	-2.7	-0.3
86975	*Mammuthus hayi*	lm3	Leisey 1A	-2.4	0.0
217737	*Mammuthus hayi*	partial molar	Leisey 1A	-2.5	-0.4
217738	*Mammuthus hayi*	partial molar	Leisey 1A	-3.8	-2.5
217739	*Mammuthus hayi*	partial molar	Leisey 1A	-2.3	-2.2
63902	*Mylohyus fossilis*	RM3	Leisey 1A	-12.2	0.5
63903	*Mylohyus fossilis*	lp4	Leisey 1A	-4.9	-0.4
67068	*Mylohyus fossilis*	RM3	Leisey 1A	-10.5	1.7
67184	*Mylohyus fossilis*	RM3	Leisey 1A	-9.5	1.3
81312	*Mylohyus fossilis*	premolar	Leisey 1A	-10.3	-3.5
84578	*Mylohyus fossilis*	Rp4	Leisey 1A	-6.7	-1.7
84753	*Mylohyus fossilis*	RP3	Leisey 1A	-10.5	-1.0
87768	*Mylohyus fossilis*	lp3	Leisey 1A	-7	-1.2
87778	*Mylohyus fossilis*	rm3	Leisey 1A	-5.8	1.4
64213	*Odocoileus virginianus*	upper molar	Leisey 1A	-12.3	-1.9

80059	*Odocoileus virginianus*	lm3	Leisey 1A	-12.8	2.8
84407	*Odocoileus virginianus*	RM	Leisey 1A	-10.3	-0.1
86377	*Odocoileus virginianus*	LP4	Leisey 1A	-10.4	2
87891	*Odocoileus virginianus*	RM3	Leisey 1A	-11.9	0.1
87906	*Odocoileus virginianus*	lm3	Leisey 1A	-10	2.7
87907	*Odocoileus virginianus*	RM3	Leisey 1A	-12.8	-0.1
64293	*Palaeolama mirifica*	RM3	Leisey 1A	-11.9	-0.5
64294	*Palaeolama mirifica*	RM3	Leisey 1A	-12.9	-0.7
64309	*Palaeolama mirifica*	RM3	Leisey 1A	-14.3	-0.2
64312	*Palaeolama mirifica*	RM3	Leisey 1A	-13.1	-0.9
64313	*Palaeolama mirifica*	RM3	Leisey 1A	-12.9	-0.4
217741	*Palaeolama mirifica*	rm3	Leisey 1A	-12.9	-0.6
63922	*Platygonus vetus*	lm3	Leisey 1A	-8	-0.1
80117	*Platygonus vetus*	lm3	Leisey 1A	-3.9	1
81238	*Platygonus vetus*	rm3	Leisey 1A	-7.4	0.9
83122	*Platygonus vetus*	rm3	Leisey 1A	-5.1	-1.2
83384	*Platygonus vetus*	RM2	Leisey 1A	-6.5	-3.8
86948	*Platygonus vetus*	LP4	Leisey 1A	-4	-2.6
87791	*Platygonus vetus*	lm3	Leisey 1A	-7.7	2.9
87830	*Platygonus vetus*	lm3	Leisey 1A	-9.2	0.8
87834	*Platygonus vetus*	RM3	Leisey 1A	-6.6	0.6
87850	*Platygonus vetus*	rm3	Leisey 1A	-3.5	2.4
65973	*Tapirus haysii*	rp2	Leisey 1A	-12	-4
85314	*Tapirus haysii*	lower molar	Leisey 1A	-12.5	-3.4
86702	*Tapirus haysii*	RP1	Leisey 1A	-12.3	-2.7
86851	*Tapirus haysii*	RM	Leisey 1A	-13.2	-4.4
86941	*Tapirus haysii*	rm	Leisey 1A	-12.8	-3.6
87941	*Tapirus haysii*	rp3	Leisey 1A	-12.5	-2.4
87957	*Tapirus haysii*	lower molar	Leisey 1A	-12.3	-4.8
88117	*Tapirus haysii*	LM	Leisey 1A	-13.3	-5.1
89533	*Tapirus haysii*	RM3	Leisey 1A	-13.4	-2.1
