# Supplementary material for: Effects of Global Warming on Ancient Mammalian Communities and Their Environments
Source: PLoS One. 2009 Jun 3;4(6):e5750. doi: 10.1371/journal.pone.0005750 (PMC2684586; doi:10.1371/journal.pone.0005750)
Supplement: Table S3 — Serial samples and descriptive statistics of Equus sp. teeth from Inglis 1A and Leisey 1A. (0.09 MB PDF) [file pone.0005750.s003.pdf]

Table S3. Serial samples and descriptive statistics of *Equus* sp. teeth from Inglis 1A and Leisey 1A.

	Inglis 1A – Glacial Site (UF 217742)			Leisey 1A – Interglacial Site (UF 85753)		
Sample ID	Distance from Crown (mm)	$\delta^{13}C_{(VPDB)}$ (‰)	$\delta^{18}O_{(VPDB)}$ (‰)	Distance from Crown (mm)	$\delta^{13}C_{(VPDB)}$ (‰)	$\delta^{18}O_{(VPDB)}$ (‰)
A	25.9	-4.4	-2.2	19.0	-2.8	1.8
B	28.5	-4.6	-3.6	21.5	-2.6	0.7
C	30.8	-4.8	-3.0	24.1	-2.7	1.1
D	33.4	-4.7	-2.8	26.4	-3.1	0.1
E	36.0	-4.3	-2.2	29.9	-2.5	1.4
F	40.0	-3.4	-1.4	32.5	-2.6	0.6
G	42.4	-2.6	-1.0	35.4	-2.2	1.9
H	45.2	-3.1	-2.2	38.0	-2.1	2.1
I	48.6	-3.7	-2.6	40.4	-2.2	1.3
J	53.5	-4.9	-1.6	43.1	-1.8	2.5
K	56.2	-4.7	-1.9	45.4	-2.0	1.2
L	-			48.2	-1.8	0.8
**Mean**		-4.1	-2.2		-2.4	1.3
**Minimum**		-4.9	-3.6		-3.1	0.1
**Maximum**		-2.6	-1.0		-1.8	2.5
**Total Range**		2.3	2.6		1.3	2.4
**SD**		0.8	0.8		0.4	0.7
