# Supplementary figures and images for: Effects of Global Warming on Ancient Mammalian Communities and Their Environments
Source: PLoS One. 2009 Jun 3;4(6):e5750. doi: 10.1371/journal.pone.0005750 (PMC2684586; doi:10.1371/journal.pone.0005750)

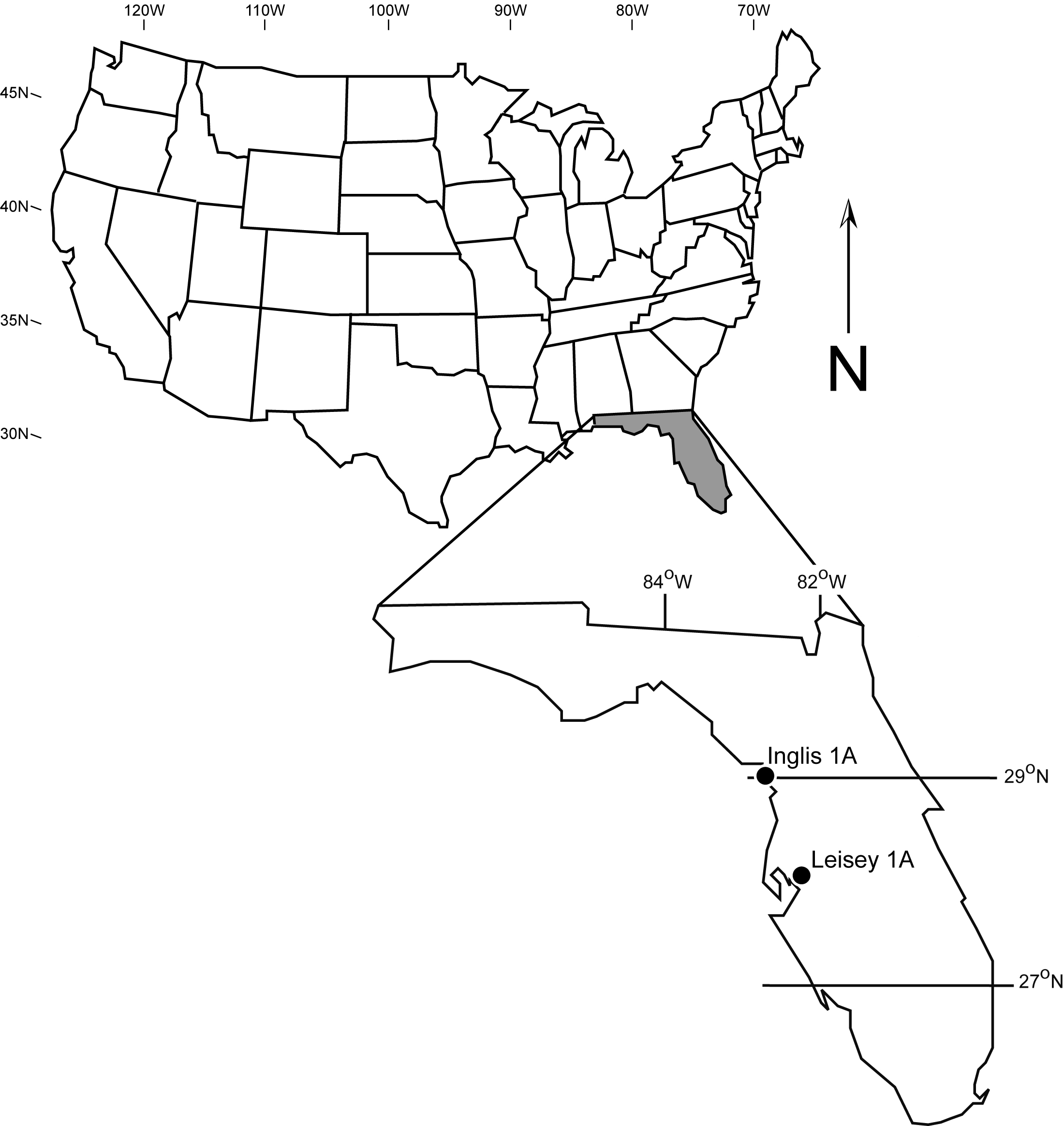

Supplement: Figure S1 — Map indicating the location of Inglis 1A and Leisey 1A in Florida, USA. (0.48 MB JPG) [file pone.0005750.s004.jpg]

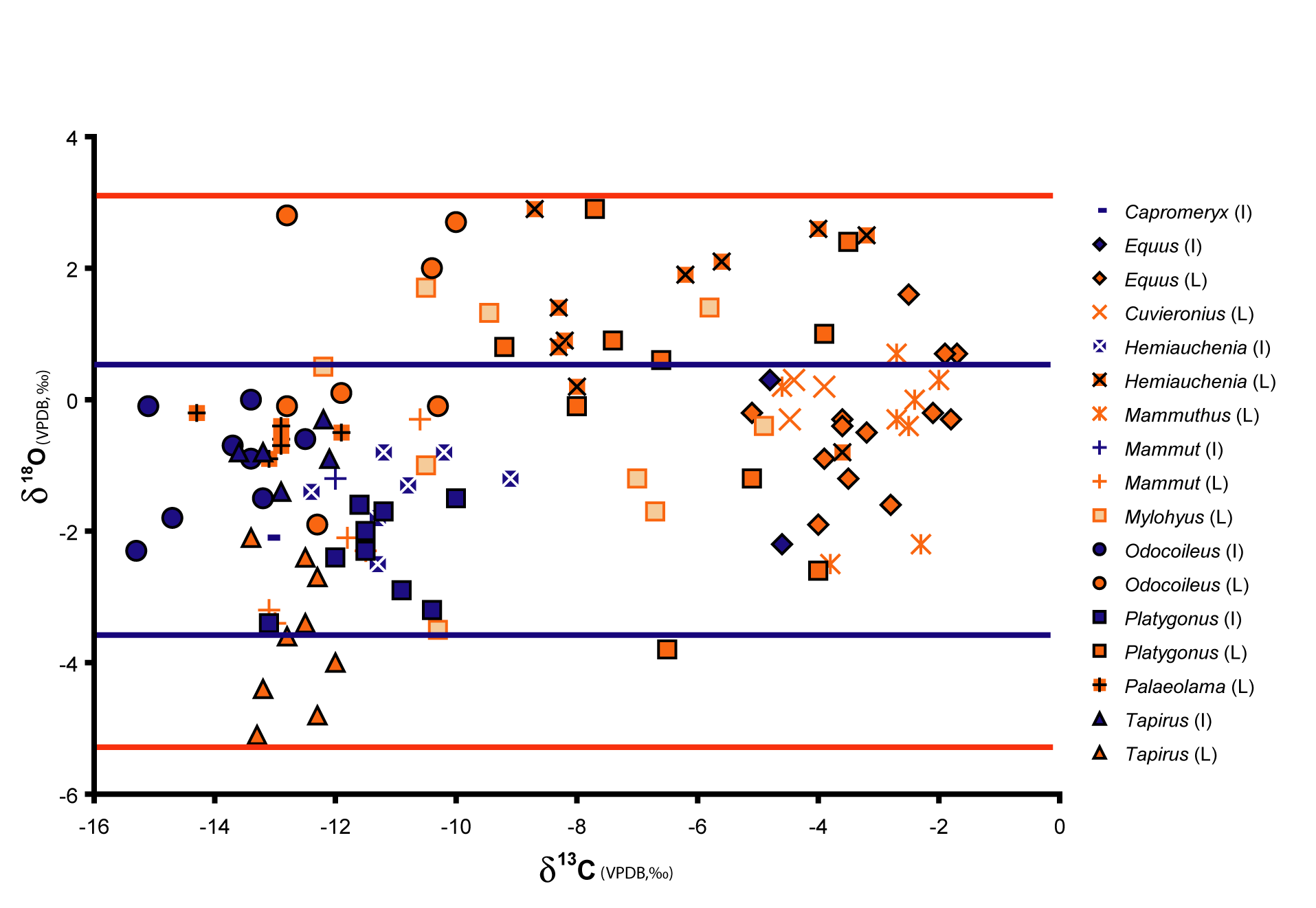

Supplement: Figure S2 — Stable carbon and oxygen isotope values for all taxa sampled. Blue symbols show taxa from the glacial fossil site Inglis 1A (I) and orange symbols show taxa from the interglacial fossil site Leisey 1A (L). The blue and orange bars indicate the total range of δ18O values for Inglis 1A and Leisey 1A, respectively. (0.31 MB TIF) [file pone.0005750.s005.tif]

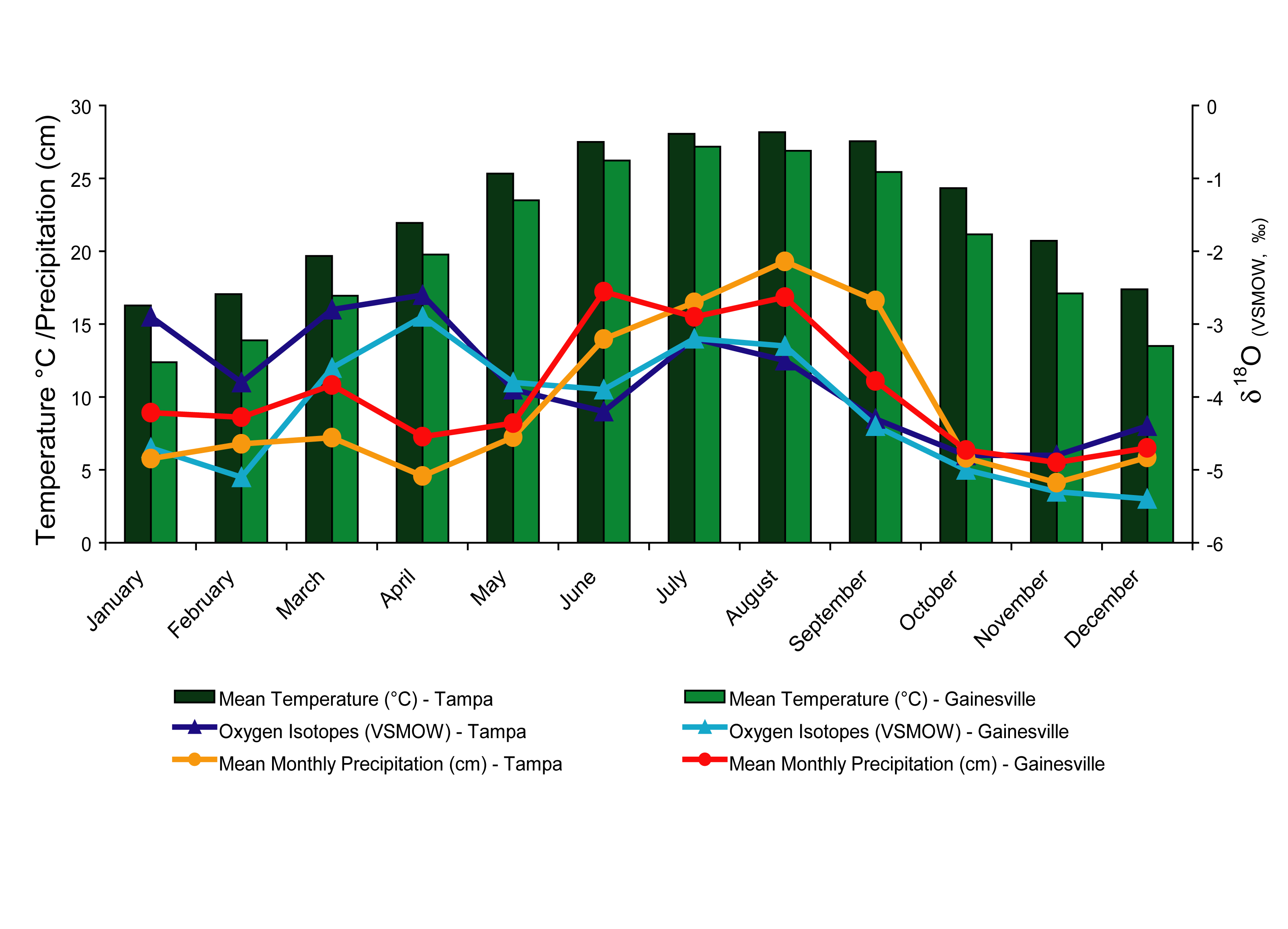

Supplement: Figure S3 — Modern climate data from Tampa and Gainesville, Florida, USA. The oxygen isotope data (rainfall precipitation) are from ISOSCAPES (www.waterisotopes.org) and temperature and precipitation data are from the National Climatic Data Center (www.ncdc.noaa.gov), with mean values from the Tampa International Airport and Gainesville Regional Airport during 1971 to 2000. (1.00 MB TIF) [file pone.0005750.s006.tif]
